# 

**Published:** 1896-02

**Authors:** 


					﻿Southern Medical Record.
A Monthly Journal of Medicine and Surgery.
Vol. XXVI. ATLANTA, GA., FEBRUARY, 1896.	No. nV
BERNARD WOLFF, M. D., Editor.	LOUIS H. JONES, M. D., Business Managed.
Associate Editors :
A. W. GRIGGS, M. D.	WILLIS F. WESTMORELAND, M. D.
j. McFadden gaston, m. d.
Enteredjat the Post Office at Atlanta, Ga., as Second-Class Matter.
The Foote & Davies Co., Atlanta, Ga.
				

